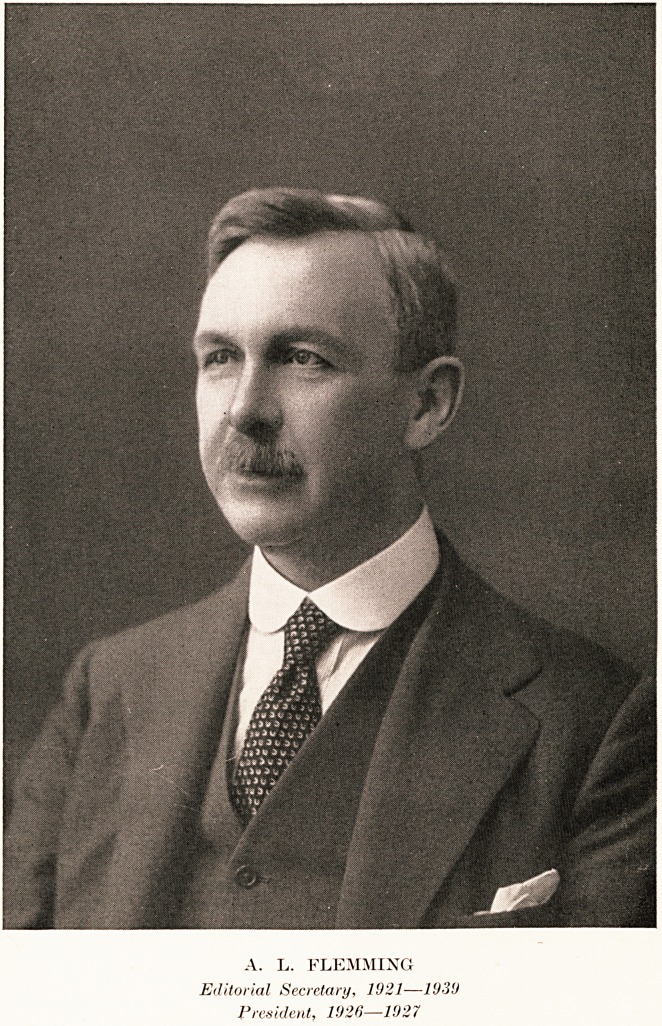# A. L. Flemming

**Published:** 1939

**Authors:** 


### Obituary

### ARTHUR LAUNCELOT FLEMMING, M.B., Ch.B.

MANY generations of past students of the Bristol Medical School, and many of the older practitioners in the district, will feel that a great gap has been left in the medical institutions of the town by the passing of one whom everybody knew, liked and respected. For how many hundreds of doctors, and to how many scores of doctors or members of their families, has he given an anæsthetic in hospital or in private? His vast experience and almost unbroken record of success gave the utmost confidence to both patient and surgeon. He was one of the pioneers, probably the leading pioneer, of ether by the open method. It was fitting that these advances should be made in the town where Humphry Davy discovered the anæsthetic properties of nitrous oxide gas. Flemming, unlike Davy, had the happiness to see his work appreciated by his professional brethren, and his methods brought into general use.

Arthur Launcelot Flemming was born in 1869, the son of Dr. Thomas H. Flemming, of Freshford, Somerset. His brother, Dr. C. E. S. Flemming, has been well known for many years by his work on the Council of the British Medical Association. He was educated at Somerset College, and Hermitage School, Bath, and studied medicine at the Bristol Medical School, qualifying in 1894. He held the posts of House Physician and of Resident Obstetric Officer at the Bristol Royal Infirmary, and later of Resident Medical Officer at the Bristol Mental Hospital. All his days he took a particular

A. L. FLEMMING
*Editorial Secretary, 1921—1939*
*President, 1926—1927*

interest in the problems of mental disease, no doubt as a result of this experience. He then commenced his life-work by accepting the post of honorary anæsthetist at the Bristol Royal Infirmary. Before long he became Lecturer in Anæsthetics, first at the Bristol Medical School and then in the University of Bristol, and although he kept together a small and select private practice, almost the whole of his work was devoted to anæsthetics.

After a good deal of experimental work he adopted as his routine for ordinary cases the administration of ether on a mask, which was a great advance on the Clover inhaler, and on chloroform anæsthesia, which previously held the field. He always believed that it is safer to keep the patient as lightly under as possible, whereas his predecessors, spurred on by impatient surgeons, were never happy until the patient was thoroughly poisoned. It is not to be denied that sometimes a word of admonition was necessary to persuade him to push the ether sufficiently for surgical comfort, but that his method was safe is beyond question. It was not until after he had finished his long years of hospital anæsthesia that a patient died on the table before the operation had begun ; it was an elderly man with myocardial degeneration, in whose case surgical treatment had been postponed again and again on account of obvious risks. He quietly remarked afterwards : "That is my first anæsthetic death." It was also the last, but I never heard him mention his record except on that one occasion.

He was President of the Anæsthetics Section of the Royal Society of Medicine for 1922–24, President of the Section of Anæsthetics at the Manchester meeting of the British Medical Association, and represented British Anæsthetics in Canada in 1923. He wrote many articles on his special subject.

He went with the Princess Christian Hospital to South Africa during the Boer War, and was in France in 1917–18 with the 56th General Hospital ; in 1914 he was at the Royal Naval Hospital, Rosyth. He was Medical Officer to the Royal School for the Blind in Bristol. He served the Bristol Medico-Chirurgical Society as President, and also as Editorial Secretary to this *Journal*.

He had many interests besides medicine. He had been a President of the Bristol Medical Dramatic Society. He was devoted to country pursuits, especially bird observation and photography and fishing. I well remember taking him out to identify a bird with an unusual song on Clifton Down : it was a garden warbler, which is not common with us. He was very

Q

thrilled when a peregrine falcon nested in the Avon Gorge. He was for one year President of the Bristol Naturalists Society.

Most of the readers of this brief notice will have their own memories of its subject. They will recall his love of the society of his fellow-men, his fund of stories, his quiet humour and readiness to laugh at a story told at his own expense. They may not know how often he had to do his work in spite of the handicap of ill-health or physical pain. For years after returning from France he was persecuted by a succession of boils and joint troubles. Times without number he had to be dissuaded from lifting the end of the theatre stretcher, because one of his ribs had fractured again. A more conscientious practitioner there never was, and the inevitable mishaps for which the anæsthetist may be blamed troubled him sore.

A very numerous and representative company gathered to do honour to his memory at the funeral service. He leaves a widow, a daughter, and son.

Dr Charles Hadfield, of London, wrote in the *British Medical Journal* :—

" All anæsthetists will have heard with real sorrow of the death on August 25th of that master of their craft, Arthur L. Flemming, of Bristol. It has been a real advantage to the science of anæsthesia in this country that, although the chief societies concerned with it naturally have their headquarters in London, some of its most distinguished practitioners have worked in the provinces and have not allowed distance to interfere with their active participation in its advancement. Many such are still with us, but to those who have passed on in the last few years, such as McCardie of Birmingham and Fairlie of Glasgow, must now be added the name of Arthur Flemming. Until failing health restricted his activities, Flemming was seldom absent from meetings of the Anæsthetic Section of the Royal Society of Medicine. It was when he was President of this Section a good many years ago that I first met him personally, and got to know him intimately as his honorary secretary. I was at once impressed by his keenness and judgement in all anæsthetic matters, whether scientific or administrative. Most of all I was impressed by the charm of his personality. In those days he travelled up from Bristol after a heavy day's work in time for a Section meeting. After it was over he and a few chosen spirits would usually assemble at my house and discuss anæsthetic affairs more freely than could be done at a public meeting. Soon it would be time for him to hurry to Paddington for his midnight train back to Bristol so as to be prepared for his work early next morning.

" Although he was never one to overvalue new methods and new agents simply because they were new, he was eager to make personal trials of them, as the result of which his considered judgement was most valuable. Concerning his skill as an administrator of anæsthetics I cannot, of course, speak from personal knowledge, but the great reputation he held with surgeons in Bristol and the West of England generally is sufficient testimonial.

" In addition to his skill as an anæsthetist he was a first-rate physician and would no doubt have made his name in this branch had his activities not been drawn to his own speciality. He was also a man of very wide interests, and I have often been impressed by the extent of his knowledge of all sorts of subjects, some of them quite outside ordinary channels of information. With all this he was modest and unassuming. Although to-day we deplore the loss of a great anæsthetist, those of us who had the privilege of his friendship mourn the passing of a really lovable colleague."

## Figures and Tables

**Figure f1:**